# Dietary Polyphenol Supplementation Prevents Alterations of Spatial Navigation in Middle-Aged Mice

**DOI:** 10.3389/fnbeh.2016.00009

**Published:** 2016-02-09

**Authors:** Julien Bensalem, Laure Servant, Serge Alfos, David Gaudout, Sophie Layé, Véronique Pallet, Pauline Lafenetre

**Affiliations:** ^1^Nutrition et Neurobiologie Intégrée, Université de Bordeaux, UMR 1286Bordeaux, France; ^2^INRA, Nutrition et Neurobiologie Intégrée, UMR 1286Bordeaux, France; ^3^Activ’InsideLibourne, France; ^4^Nutrition et Neurobiologie Intégrée, Bordeaux INP, UMR 1286Bordeaux, France

**Keywords:** age, hippocampus, berries, polyphenols, learning and memory, strategy, navigation

## Abstract

Spatial learning and memory deficits associated with hippocampal synaptic plasticity impairments are commonly observed during aging. Besides, the beneficial role of dietary polyphenols has been suggested as potential functional food candidates to prevent this memory decline. Indeed, polyphenols could potentiate the signaling pathways of synaptic plasticity underlying learning and memory. In this study, spatial learning deficits of middle-aged mice were first highlighted and characterized according to their navigation patterns in the Morris water maze task. An eight-week polyphenol-enriched diet, containing a polyphenol-rich extract from grape and blueberry (PEGB; from the Neurophenols Consortium) with high contents of flavonoids, stilbenes and phenolic acids, was then successful in reversing these age-induced effects. The use of spatial strategies was indeed delayed with aging whereas a polyphenol supplementation could promote the occurrence of spatial strategies. These behavioral results were associated with neurobiological changes: while the expression of hippocampal calmodulin kinase II *(CaMKII)* mRNA levels was reduced in middle-aged animals, the polyphenol-enriched diet could rescue them. Besides, an increased expression of nerve growth neurotrophic factor *(NGF)* mRNA levels was also observed in supplemented adult and middle-aged mice. Thus these data suggest that supplementation with polyphenols could be an efficient nutritional way to prevent age-induced cognitive decline.

## Introduction

Aging is associated with cognitive impairments and increased risks of neurodegenerative disorders such as Alzheimer’s disease which may contribute to the loss of the ability to live independently (Evans et al., 1989). In this context it seems paramount to better understand the effects of aging on learning and memory and to develop new strategies to prevent or counteract the age-associated memory decline.

The hippocampus is one of the brain structures involved in spatial learning and memory which is particularly affected during aging (Erickson and Barnes, 2003). These impairments have been observed not only in rodents (Barnes, 1979; Markowska et al., 1989; Gallagher and Rapp, 1997; Bach et al., 1999) but also in monkeys (Lai et al., 1995; Gallagher and Rapp, 1997) and in humans (Uttl and Graf, 1993; Wilkniss et al., 1997; Gazova et al., 2013). Indeed, in a real-space adapted version of the Morris water maze for humans, it has been shown that the profile of allocentric (world-centered, hippocampus-dependent) spatial navigation learning, but not egocentric navigation, is particularly altered in adults over 70 (Gazova et al., 2013). The age-related profile of spatial navigation has also been studied in mice in the star maze and in the Morris water maze (Martel et al., 2007; Fouquet et al., 2011). Although it is accepted that aged mice (over 17 months old) abandon sequential egocentric and/or allocentric (hippocampus-dependent) strategies in favor of egocentric or cued (striatum-dependent) strategies (Kim et al., 2001; Martel et al., 2007), the training protocols (massed vs. spaced) may influence the adoption of a strategy over another. Indeed, these studies cannot fully translate the parallel and progressive acquisition of non-spatial to spatial strategies that are observed in the Morris water maze (Janus, 2004; Brody and Holtzman, 2006; Stone et al., 2011; Ruediger et al., 2012), and that depends on the interaction of multiple memory systems (Gazova et al., 2013). It is thus of interest to further analyze how aging may influence this evolution (Gil-Mohapel et al., 2013) and the differential use of non-spatial and spatial strategies by middle-aged and aged animals. The neurobiological basis for differences in navigation patterns is not fully understood but based on previous studies it is possible to hypothesize that differential activations of the hippocampus and the striatum during the learning phase could explain this evolution. Indeed, the hippocampus and the striatum must act in parallel during the acquisition of the task (Colombo et al., 2003; Martel et al., 2007).

Long-term memories are associated with molecular changes (Carew, 1996; Kandel, 2001; Sossin, 2008), such as the synthesis of new mRNAs and proteins (Martin et al., 2000; Kelleher et al., 2004; Bramham and Wells, 2007). Various signaling pathways involved in the control of *de novo* protein synthesis such as extracellular signal regulated kinase (ERK) (Sweatt, 2001, 2004) converge to the activation of the transcription factor cAMP-response element-binding protein (CREB) which binds the promoter regions of many genes associated with synaptic plasticity underlying spatial learning and memory (Harris and Kater, 1994; Impey et al., 1996, 1998, 2004; Pham et al., 1999; Barco et al., 2006; Alvarez and Sabatini, 2007). Hippocampal levels of phosphorylated calmodulin kinase II (CaMKII), known to regulate the phosphorylation of CREB (Dash et al., 1991), are reduced in aged animals relative to young animals. CREB also regulates the transcription of neurotrophins, such as brain derived neurotrophic factor (BDNF) or nerve growth neurotrophic factor (NGF), implicated not only in neuronal survival, outgrowth and differentiation (Finkbeiner et al., 1997; Finkbeiner, 2000; Schinder and Poo, 2000; Conkright et al., 2003; Pruunsild et al., 2011) but also in the control of synaptic plasticity and long-term memory (Poo, 2001; Bramham and Messaoudi, 2005; Calabrese et al., 2009). Similarly, the use of non-spatial strategies could trigger their expression in the striatum.

There is increasing evidence of how dietary habits or nutrients may exert beneficial effects on brain aging. Among “functional foods” promoting a healthy aging, fruits and vegetables rich in polyphenols are now highly studied for their potential beneficial effects on memory (Shukitt-Hale et al., 2008; Rendeiro et al., 2009; Kean et al., 2015). Within the polyphenol family, flavonoids have been shown to ameliorate learning and memory in both animals and humans. Flavanols, and particularly epicatechin and catechin, present in grape seeds, green tea or cocoa for example, have been shown to ameliorate learning and memory in animals (Devi et al., 2006; Haque et al., 2006; van Praag et al., 2007; Kaur et al., 2008; Asha Devi et al., 2011; Rendeiro et al., 2013b) and in humans (Dinges, 2006). Anthocyanins, present in red berries as in blueberries, have also been shown to prevent memory deficits in aged animals (Cho et al., 2003; Ramirez et al., 2005; Barros et al., 2006; Shukitt-Hale et al., 2006; Rendeiro et al., 2013b). About flavanols, quercetin has been undoubtedly the most extensively studied flavonol in their neuroprotective potential *in vivo* (Dajas et al., 2003, 2013; Rivera et al., 2004; Ahmad et al., 2011). Clinical studies have also observed an improvement of memory in older people with a supplementation with grape juice or with blueberry juice rich in flavonoids (Krikorian et al., 2010a,b, 2012). Moreover, flavonoid consumption has been associated with better cognitive performance in an epidemiological study over 10 years (Letenneur et al., 2007). Stilbenes and more precisely resveratrol, found mainly in grapes and wine, have displayed beneficial effects on learning and memory in animals (Abraham and Johnson, 2009; Dal-Pan et al., 2011; Harada et al., 2011; Kodali et al., 2015) and in humans (Witte et al., 2014). A naturally dimethylated analog of resveratrol, pterostilbene found in blueberries (Aiyer et al., 2012) and grapes (Adrian et al., 2000), exhibits similar biological activities (Rimando et al., 2002) and have been reported to be effective in reversing cognitive deficits in aged rats (Joseph et al., 2008). Phenolic acids are a very large polyphenols family as most of them can be the results of the microbial metabolism of other polyphenols (Margalef et al., 2014). Phenolic acids have also displayed beneficial effect to reverse memory impairments (Yan et al., 2001; Vauzour et al., 2007). Interestingly, polyphenols are not only able to impact on learning and memory during aging but they can also modulate neuronal signaling cascades altered with aging. Thereby, polyphenols may act on ERK/CREB pathway involved in synaptic plasticity and long-term potentiation (Williams and Grayer, 2004; Schroeter et al., 2007; Vauzour et al., 2007; Rendeiro et al., 2009). Indeed, flavonoid supplementations can modulate specific signaling kinases like CaMKII (Abraham and Johnson, 2009; Rendeiro et al., 2009), and ERK (Rendeiro et al., 2009, 2013a), controlling the activation of CREB and the increased expression of BDNF (Abraham and Johnson, 2009; Rendeiro et al., 2009, 2013a; De Nicoló et al., 2013) and of NGF (De Nicoló et al., 2013) in the hippocampus. Some studies have also reported that polyphenols, among them flavanols, anthocyanins or resveratrol, are found in the brain tissue after oral ingestion (Abd El Mohsen et al., 2002, 2006; Asensi et al., 2002; Andres-Lacueva et al., 2005; Williams et al., 2008; Prasain et al., 2009; Juan et al., 2010; Milbury and Kalt, 2010). The possible existence of specific polyphenol binding sites at the cellular plasma membrane level in the rat brain has been suggested (Han et al., 2006).

While flavonoids and stilbenes, present in berry extracts, have potential properties against age-related cognitive decline, their specific benefits on spatial navigation patterns are unknown.

In this study, we investigated the effects of age and of a polyphenol-rich extract from grape and blueberry (PEGB; from the Neurophenols Consortium) supplementation, with high contents of flavonoids, on hippocampus-dependent learning and memory processes. First, the learning and memory deficits observed in middle-aged mice were better characterized according to their navigation patterns in the Morris water maze, then, the effectiveness of dietary polyphenols in the prevention of age-associated memory decline was assessed and the neurobiological correlates underlying these effects were investigated.

## Materials and Methods

### Animals and Diet

**Figure 1:**
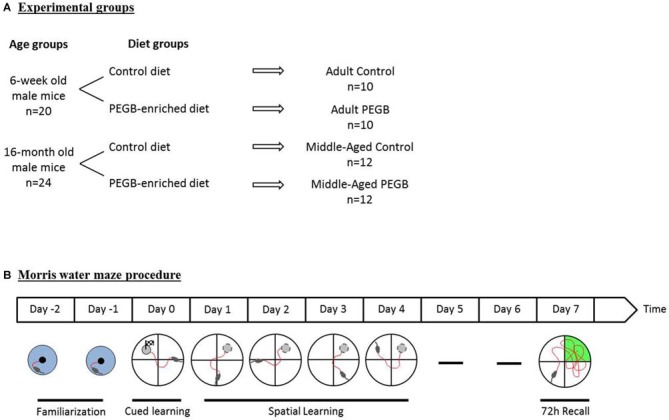
**Experimental design. (A)** Six-week old and sixteen-month old mice were either fed a control diet or a polyphenol-enriched diet (500 mg of PEGB/kgbw/day) for 8 weeks (i.e., 6 weeks before performing the Morris water maze test). **(B)** A 2-day familiarization period was performed before the 1-day cued learning. Spatial learning has then been evaluated over 4 days. Seventy two hours after the last learning trial a probe test was performed in order to evaluate spatial memory.

For this study 20 adult (6-week old) and 24 middle-aged (16-month old) male C57Bl/6J mice were purchased from Janvier (France). They were singly housed in a room with a constant airflow system, controlled temperature (21–23°C), and a 12 h light/dark cycle. Mice were given *ad libitum* access to food and water. Naïve mice were randomly divided into four experimental groups (Figure 1A). One group of adult mice (*n* = 10) and one of middle-aged mice (*n* = 12) were fed with a control diet (INRA Jouy-en-Josas, France), whereas the two other groups of adult mice (*n* = 10) and middle-aged mice (*n* = 12) received a PEGB-enriched diet (INRA Jouy-en-Josas, France) containing 500 mg of PEGB/kgbw/day (provided by the Neurophenols Consortium). The composition of the PEGB-enriched pellets was the same as the control pellets, except for the polyphenol content (Table 1). Diets started as soon as mice arrived in the laboratory (i.e., at the age of 6 weeks for adult mice and 16 months for middle-aged mice) and continued throughout the entire experiment (8 weeks). Thereby, at the end of the experiment adult mice were 3.5 months old and middle-aged mice were 18 months old. The PEGB is a powder made of grape (*Vitis vinifera* L.; Activ’Inside, France) and blueberry (*Vaccinium angustifolium*; NutraCanada, Canada) extracts containing specific polyphenols with low molecular weight (flavanols monomers and oligomers, flavonols, anthocyanins, phenolic acids and resveratrol) formulated in a unique ratio of molecules. The 500 mg of PEGB/kgbw/day dose was determined by a literature review and proportions for each polyphenol class of interest were adjusted in the mix. This specific ratio of polyphenols remains confidential. All experiments were performed in accordance with the European Communities Council Directives (86/609/EEC) and the French national Committee (87/848) recommendations, and have been approved by the Animal Care and Use Committee of Bordeaux under the N°5012085-A.

**Table 1:** **Composition of the control and the PEGB-enriched diets**.

	Percent (%)	
Components	Control diet	PEGB-enriched diet
Hydrochloric casein	18	18
Corn starch	40	45
Sucrose	29.9	24.39
Cellulose	2	2
Peanut oil	2.5	2.5
Rapeseed oil	2.5	2.5
Mineral coumpoud 102	4	4
Vitamin coumpoud 102	1	1
without vitamine A	–	–
+ DL methionine	0.1	0.1
+ Vitamin A 5 UI/g	5UI/g	5UI/g
PEGB	0	0.51

*PEGB extract was introduced at 0.51% in the diet pellets*.

### Body Weight and Food Intake

Body weight and food intake were monitored weekly during the 8-week supplementation period.

### Spatial Learning and Reference Memory in the Morris Water Maze

#### Training Phase

Six weeks after the beginning of the diets, spatial learning and memory were assessed in a Morris water maze (150 cm in diameter, 50 cm-high) filled with white water (22°C) and surrounded with distal extramaze cues (Figure 1B). Before being trained, animals were handled 1 min a day for 2 days. Mice were then familiarized to water and swimming during two familiarization days (day-1 and day-2) where they had to find a visible platform in the center of a small pool (60 cm diameter) surrounded with curtains (three consecutive trials a day; 60 s-cut-off). On day 0, to evaluate visuomotor deficits, mice were given six trials (90 s-cut-off) to find a visible platform pointed out with a cue in the Morris water maze that was surrounded with white curtains. During the training sessions (days 1–4), animals were required to locate the submerged platform by using distal extramaze cues. They were trained for six trials a day (90 s-cut-off) with an intertrial interval of 5 min for four consecutive days. In order to facilitate spatial learning, mice were introduced from four different starting points, in a randomized daily order. The speed, the latency and the distance to reach the platform as well as the swim path of each trial were recorded by Imetronics videotracking system (France). The daily swim path efficiency was calculated as the ratio of the shortest possible length to the effective swim path length.

#### Probe Test

Seventy two hours after the last training session, the platform was removed from the pool and spatial memory was evaluated for 60 s. The percentage of distance traveled in the four quadrants was recorded using the SMART system (San Diego Instruments). The quadrant where the platform was located during training is referred to as target quadrant. Additionally, the number of annulus crossings and the mean proximity to the platform were assessed during this test as reliable measures of probe test performance (Maei et al., 2009).

### Analysis of Navigation Strategies

For each trial of the training phase, the navigation path was analyzed from the replay of the videotracking system (Imetronics, France) and assigned to one of the eleven strategies by two experimenters, blind to the groups. The categorization scheme (see Figure 4A) was adapted from those developed previously (Brody and Holtzman, 2006; Garthe et al., 2009; Stone et al., 2011; Ruediger et al., 2012). These strategies were divided into two main categories: non-spatial vs. spatial strategies. *Non-spatial strategies* included first “global search” strategies: “peripheral looping” (persistent swimming around the outer 15 cm of the pool, including thigmotaxis), “random” (searching the entire tank, >75% surface coverage), “circling” (swimming in tight circles, possibly with some net directional movement), and then “local search” strategies: “scanning” (searching restricted to a limited, often central, portion of the tank, >15% and <75% of surface coverage), “chaining” (circular swimming at an approximately fixed distance greater than 15 cm from the wall), “repeated incorrect” (swimming in a precise direction that does not contain the platform and repeat the same trajectory several times), and “focal incorrect” (searching intently a small portion of the tank that does not contain the platform). *Spatial strategies* included “repeated correct” (swimming in direction of the platform and repeat the same trajectory several times), “focal correct” (swimming and searching intently in the zone containing the platform), “spatial indirect” (swimming indirectly to the platform with eventually 1–2 loops) and “spatial direct” (swimming directly to the platform).

### Tissue Preparation

Ninety minutes after the probe test, mice were euthanized by cervical dislocation and decapitated. Brains were rapidly removed and hippocampi were dissected and frozen with liquid nitrogen before being stored at −80°C until assay.

### Quantitative Real-Time PCR Analysis

Hippocampal and striatal gene expression were measured as previously described (Touyarot et al., 2013). RNA extraction was conducted using TRIzol reagent kit (Invitrogen, France) according to the manufacturer’s instructions. The integrity of the purified RNA was verified using the RNA 6000 Nano LabChip kit in combination with the 2100 Bioanalyzer (Agilent Technologies, France). The concentrations of RNA were determined by using a Nanodrop ND-1000 (Labtech, France). Using oligodT and random primers (Promega, France), cDNA was synthesized from 1 μg of RNA with ImPromII reverse transcriptase (Promega, France) according to the manufacturer’s protocol. Briefly, 1 μg of total RNA mixed with RNasin (Promega) and DNase (Roche) was incubated at 37°C. Then, OligodT and random primers were added for incubation at 70°C. The reverse transcriptase reaction was performed at 42°C for 60 min in a final volume of 20 μl.

The real-time PCR was performed using the LightCycler 480 system with a 96-well format (Roche Diagnostics, Germany) in a volume of 20 μL, containing 1 × LightCycler 480 SYBR Green I Master solution, 0.5 μM of each primer and 6 μL of cDNA. The forward and reverse primer sequences and the amplicon size *Actin, NGF, BDNF, CaMKII, ERK1* and *ERK2* are summarized in Table 2. Actin was used as the reference gene since its expression level was unaffected in our experimental conditions. The following program started with an initial denaturation step for 10 min at 95°C, then an amplification for 40 cycles (10 s denaturation at 95°C, 6 s annealing at 62°C, and 10 s extension at 72°C), finally a melting curve analysis was run. In order to verify the specificity and the identity of the amplified products: (1) the melting curve analysis showed a single melting peak after amplification; and (2) amplified products for each gene were verified by sequencing with the Big Dye Terminator v1.1. (Applied Biosystems) and analyzed on a ABI3130 sequencer (Applied Biosystems).

**Table 2:** **Forward and reverse primer sequences and amplicon size used for LightCycler RT-qPCR**.

Gene name	Nucleotide sequence 5′-3′	Amplicon size (bp)
*Actin*	F: AAAACGCAGCTCAGTAACAGTCC R: AGGATGCAGAAGGAGATTACTGC	220
*NGF*	F: ATCAAGGGCAAGGAGGTGACAG R: GAGTTCCAGTGTTTGGAGTCGATG	143
*BDNF*	F: AACCATAAGGACGCGGACTTG R: TTGACTGCTGAGCATCACCC	51
*Erk1*	F: TCCCCATAGCCTGAGTGATGAG R: CCATTCCAGAACGGTCTACCAGA	102
*Erk2*	F: TTCCCAAATGCTGACTCCAAAG R: AAGTCGTCCAACTCCATGTCAAAC	179
*CaMKII*	F: AGATGTGCGACCCTGGAATGAC R: AGTGATGCGGATATAGGCGATGC	194

*Actin; NGF, Nerve Growth Factor; BDNF, Brain-Derived Neurotrophic Factor; ERK, Extracellular signal-Regulated Kinases; CaMKII, Calmodulin-dependent protein kinase II*.

Quantification data were analyzed using the LightCycler 480 Relative Quantification software (version 1.5). In order to compensate for differences in target and reference gene amplification efficiency, either within or between experiments, this software provides a calibrator-normalized relative quantification including a PCR efficiency correction. Therefore, the results are expressed as the target/reference ratio divided by the target/reference ratio of the calibrator. In our case, the calibrator was chosen among the adult mice.

### Statistical Analysis

Statistical analyses were performed using StatView^®^ (SAS Institute Inc., USA). Graphical representations were performed using GraphPad Prism^®^. Results were considered significantly different when *p* < 0.05. All results are expressed as mean ± SEM. Food intake, body weight gain, probe test comparisons (such as the traveled distance in target quadrant, the swim speed during the cued task and the probe test, the platform annulus crossings and the proximity to the platform) and PCR data were analyzed using a 2-way ANOVA (with two factors: age and diet) followed by a *post hoc* Fisher PLSD test when appropriate. Spatial learning, search strategy, swim speed during the learning phase, swim path efficiency, probe test analysis were analyzed using a 3-way ANOVA with repeated measures (age, diet and days or quadrants) followed by a *post hoc* Fisher PLSD test when appropriate. Probe test comparisons of each group with chance level (25%) were performed with a one-sample *t*-test. For the distance traveled in different quadrants during the probe test, intra-group comparisons were performed using a one-way ANOVA followed by a Dunnett’s multiple comparison test.

During the cued phase of the behavioral task, one middle-aged control mouse was excluded from the experiment because it was floating and did not look for the platform. During the training phase, five mice (one adult control mouse, two adult supplemented mice, one middle-aged control mouse and one middle-aged supplemented mouse) were excluded from the experiment because they may not have learned that there was an escape platform, therefore they were not searching for it. They were identified thanks to an intra-group outlier analysis on the distance and the latency to reach the platform (mean ± 2 SD).

## Results

### Effects of Age and PEGB-Enriched Diet on Food Intake and Weight Gain

**Figure 2:**
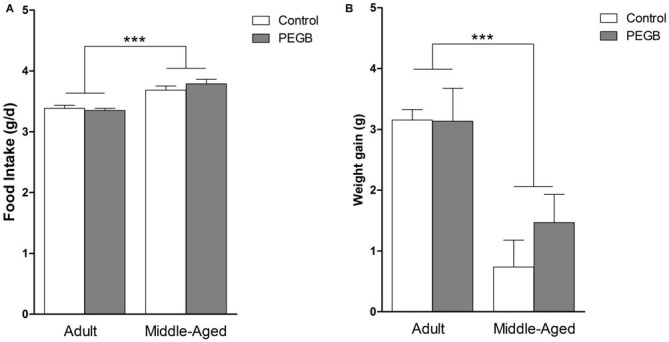
**Effects of 8 weeks of PEGB supplementation on food intake and body weight gain. (A)** Aging increases food intake but PEGB does not have any impact on it (age effect ***p < 0.0001 by 2-way ANOVA; *n* = 9–11 per group). **(B)** Adult mice gain more weight during the 8-week supplementation than middle-aged mice but PEGB does not impact on their weight gain (age effect ***p < 0.0001 by 2-way ANOVA; *n* = 9–11 per group).

Food intake and weight gain were measured along the 8 weeks of diet exposure. Middle-aged mice consume more food than adult mice whatever the diet as revealed by the 2-way ANOVA on average food intake (effect of age [*F*_(1,32)_ = 84.451, *p* < 0.0001], no effect of diet [*F*_(1,32)_ = 0.724, n.s.] and no age × diet interaction [*F*_(1,32)_ = 12.387, n.s.]) (Figure 2A).

Adult mice that are completing their full growth gain more weight over the 8 weeks than middle-aged mice regardless of the diet. Indeed, a 2-way ANOVA on average body weight gain revealed an age effect (*F*_(1,32)_ = 20.958, *p* < 0.0001), no diet effect (*F*_(1,32)_ = 1.587, n.s.), and no age × diet interaction (*F*_(1,32)_ = 0.007, n.s.) (Figure 2B).

### Effects of Age and PEGB-Enriched Diet on Spatial Learning and Memory

**Figure 3:**
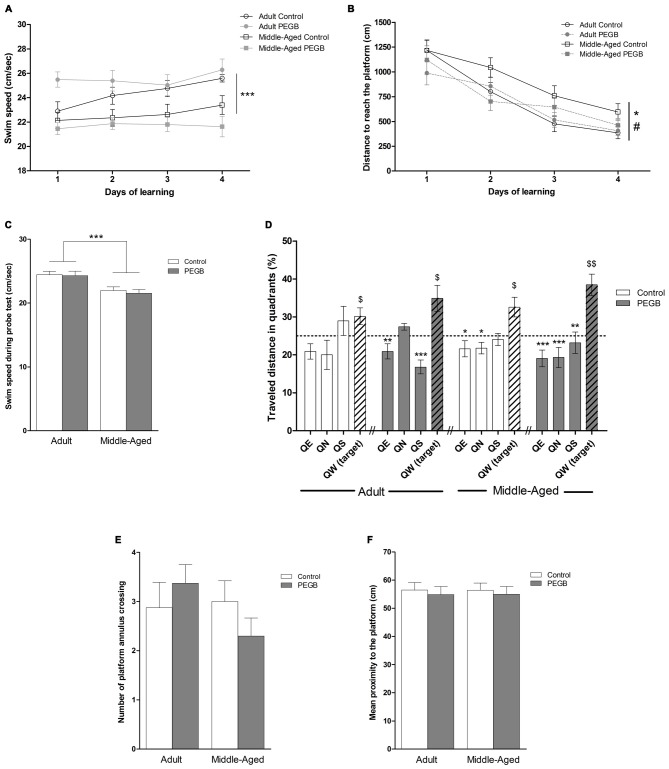
**Effects of 8 weeks of PEGB supplementation on spatial learning and memory. (A)** Swim speed during learning is reduced with aging but not affected by the supplementation with PEGB (age effect: ***p < 0.001 by 3-way ANOVA; *n* = 9–11 per group). **(B)** Distance covered to reach the hidden platform over the four consecutive days of spatial learning (trials are averaged for each training day). Middle-aged mice exhibit longer distance compared to adult mice to reach the platform during the training sessions while mice fed with the PEGB-enriched diet travel less distance to reach the platform than mice under a control diet (day effect *p* < 0.0001; age effect *p < 0.05; diet effect ^#^*p* < 0.05 by 3-way ANOVA with repeated measures). **(C)** Swim speed during the probe test is reduced in middle-aged mice (age effect: ***p < 0.0001). **(D)** Percentage of distance traveled in quadrants during the probe test. The dotted line corresponds to chance level (25%). All groups can remember the platform location traveling preferentially more distance in the target quadrant and the PEGB-enriched diet slightly ameliorates memory performance ($*p* < 0.05, $$*p* < 0.01 vs. chance level by one sample *t*-test. *p < 0.05, **p < 0.01, ***p < 0.001 compared to QW-Target by One-way ANOVA and Dunnett’s multiple comparison test; *n* = 9–11 per group). **(E)** The number of crossings of the platform annulus is not impacted by age nor by PEGB. **(F)** The mean proximity to the platform is similar for all groups.

To acquire the procedural aspects of the task such as swimming and climbing onto the platform and to evaluate visuo-motor capabilities, mice were first trained to find a visible platform in the Morris water maze without distal cues. Middle-aged mice swam slower than adult mice, under a control or a PEGB-enriched diet as revealed by an ANOVA performed on the swim speed over the trials in the cued task (age effect [*F*_(1,32)_ = 4.325, *p* < 0.05], with no effect of diet [*F*_(1,32)_ = 0.354, n.s.] and no interaction age × diet [*F*_(1,32)_ = 0.959, n.s.]) (data not shown). The same observation has been made during the learning phase (age effect [*F*_(1,32)_ = 17.758, *p* < 0.001], with no effect of diet [*F*_(1,32)_ = 0.089, n.s.], no interaction age × diet [*F*_(1,32)_ = 1.642, n.s.] and no day effect [*F*_(1,96)_ = 2.648, n.s.]) (Figure 3A). As the latency to reach the platform is dependent on the swimming speed, the distance covered to reach the platform was chosen as a more appropriate measure to show the acquisition rate for this cued task and for spatial learning.

In this way, all groups had similar visual capabilities and did not show any impairment in the cued version of the Morris water maze task. Indeed, all groups traveled similar distances to reach the visible platform because the 2-way ANOVA on the distance to reach the visible platform indicated no effect of age (*F*_(1,32)_ = 2.935, n.s.), no effect of diet (*F*_(1,32)_ = 0.012, n.s.), and no age × diet interaction (*F*_(1,32)_ = 0.905, n.s.) (data not shown).

From day 1, mice were trained in the spatial version of the Morris water maze to test the effects of age and PEGB on spatial learning and memory. The four groups of mice traveled significantly less and less distance over the 4 days (Figure 3B), indicating that all groups learned the platform location (day effect [*F*_(3,96)_ = 39.862, *p* < 0.0001]). However, middle-aged mice traveled significantly longer distance than adult mice to find the platform, revealing spatial learning deficits (ANOVA: age effect [*F*_(1,32)_ = 5.256, *p* < 0.05]). Moreover, mice that are under a PEGB-enriched diet showed better performance than mice under a control diet (ANOVA: diet effect [*F*_(1,32)_ = 4.182, *p* < 0.05]). Interestingly, there was no interaction age × diet, suggesting that this diet effect was observed not only in middle-aged supplemented mice which performed as well as adult control mice (ANOVA [*F*_(1,32)_ = 2.137, n.s.]), but also in adult mice that are under the PEGB-enriched diet and seem to perform better especially on the first day of training. On the last training day, all groups traveled similar distance to reach the platform (Figure 3B). These results were confirmed by the analyses on the swim path efficiency. Indeed, the ANOVA similarly revealed a day effect (*F*_(1,96)_ = 44.837, *p* < 0.0001) an age effect (*F*_(1,32)_ = 8.551, *p* < 0.01) and a trend for an interaction age × diet (*F*_(1,32)_ = 3.976, *p* = 0.0547) and for a diet effect (*F*_(1,32)_ = 3.206, *p* = 0.0828) (Supplementary Figure 1). Indeed, on the first learning trials, all mice may complete the task by chance (as they have not constructed any cognitive map yet): this could hide the effects of polyphenols in the path efficiency.

Spatial memory was assessed during the probe test. As the swim speed is still affected by age on this trial (Figure 3C), the traveled distance has been chosen as the most appropriate parameter. One sample test comparing to the chance level (25%) revealed that all groups swam significantly more distance in the target quadrant (adult control: *p* < 0.05; adult supplemented: *p* < 0.05; middle-aged control: *p* < 0.05; middle-aged supplemented: *p* < 0.01) (Figure 3D), revealing that 72 h after the last training session, the four groups remembered the location of the platform. Besides, a 2-way ANOVA performed on the percentage of distance traveled in the different quadrants revealed no quadrant × age interaction (*F*_(1,96)_ = 0.395, n.s.) but a quadrant × diet interaction (*F*_(1,96)_ = 3.513, *p* < 0.05), suggesting that PEGB may improve spatial memory. A one-way ANOVA realized for each group revealed a quadrant effect for the supplemented adult mice (*F*_(1,32)_ = 9.465, *p* < 0.001), the control middle-aged mice (*F*_(1,32)_ = 5, 051, *p* < 0.01) and the supplemented middle-aged mice (*F*_(1,32)_ = 9.532, *p* < 0.001). Surprisingly, no quadrant effect was revealed for the adult mice under a control diet (*F*_(1,32)_ = 2.244, n.s.) as they preferentially traveled within two adjacent quadrants (the target west and the south quadrants). However, as previously described, adult control mice traveled more than 30% of the total distance in the target quadrant. However, according to additional analyses no differences were revealed by the between-group ANOVA for the number of annulus crossings and the mean proximity to the platform (Figures 3E,F). Thus, the PEGB-enriched diet only slightly improves memory performance in the spatial version of the Morris water maze.

### Effects of Age and PEGB-Enriched Diet on Strategies During Spatial Learning

**Figure 4:**
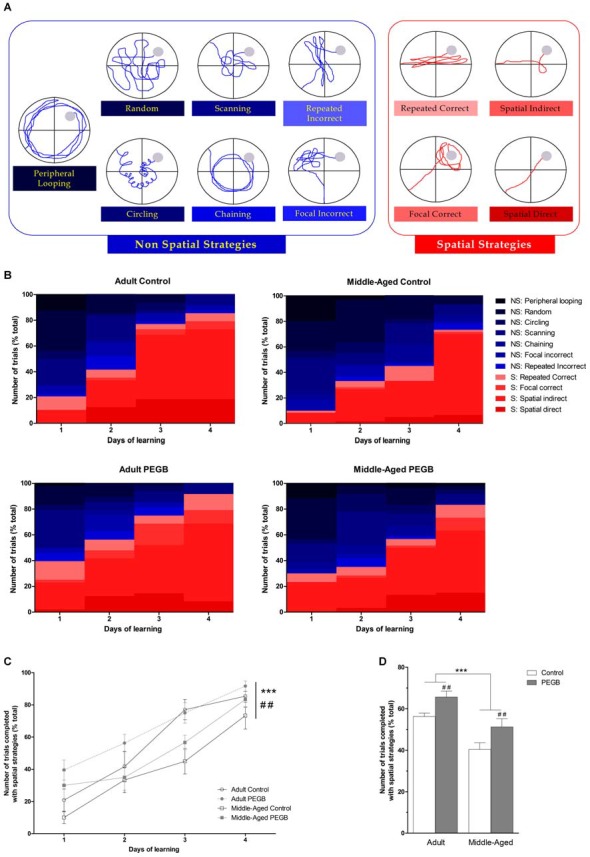
**Effects of 8 weeks of PEGB supplementation on search strategies during spatial learning in the Morris water maze. (A)** Representative path patterns that reflect “non-spatial” (blue) or “spatial” (red) strategies used to reach the hidden platform (filled gray circle). Operational definitions of individual strategies are described in the method section. **(B)** Search strategies used during learning. Non-spatial strategies are represented in blue colors and spatial strategies in red colors. **(C)** All groups show an evolution of the use of non-spatial to spatial strategies (day effect *p* < 0.0001). An age effect and a diet effect are also observed (age effect: ***p < 0.0001; diet effect: ^##^*p* < 0.01). **(D)** Adult mice exhibit more use of spatial strategies than middle-aged mice. Moreover mice fed with the PEGB-enriched diet performed more spatial strategies than mice under the control diet (age effect: ***p < 0.0001; diet effect: ^##^*p* < 0.01 by 3-way ANOVA; *n* = 9–11 per group).

In order to better characterize the age-induced spatial learning deficits, revealed with the conventional parameters, and whether dietary polyphenols could be beneficial, the navigation path of each trial was qualitatively analyzed (Figures 4A,B). The detailed analysis of navigation path revealed that control mice progressively applied different search strategies along the learning phase. Usually, consistent with previous reports (Janus, 2004; Ruediger et al., 2012), mice first adopt non-spatial strategies, beginning with global search (random swim) and then local search strategies (scanning and chaining). The strategies “focal incorrect” and “repeated incorrect” categorized as non-spatial strategies are in fact intermediate, suggesting that mice may use distal cues in an erroneous manner and that their cognitive map is not fully acquired. Then, spatial strategies (repetition of a direct path, indirect search, focal search, direct swim) appear. However, it is of note that control mice do not always go through all strategies. The increase of the use of spatial strategies evolves continuously but not linearly over the trials and correlates with a decreased average distance to reach the platform.

An evolution from non-spatial to spatial strategies over the learning days of the four groups is observed. Indeed, more and more trials are solved using one of the four spatial strategies (3-way ANOVA on navigation strategies: day effect [*F*_(3,96)_ = 51.242, *p* < 0.0001]) (Figure 4C). Moreover, this analysis revealed an age effect (*F*_(1,32)_ = 23.116, *p* < 0.0001) and a diet effect (*F*_(1,32)_ = 10.345, *p* < 0.01). These data suggest that middle-aged mice take more time to adopt spatial strategies than adult mice (only 50% of middle-aged control mice and 60% of middle-aged supplemented used a spatial strategy on the first day) and that mice under the PEGB-enriched diet are more prone to use spatial strategies than mice under the control diet. This diet effect is principally observed in the first learning day for adult mice where an ANOVA revealed a significant diet effect (*F*_(1,32)_ = 8.114, *p* < 0.001) (Figure 4C): indeed, 100% of the adult mice consuming PEGB were able to use one of the spatial strategies within the six first trials whereas only 62.5% of adult mice under the control diet did so (data not shown).

This is confirmed when all trials over the four learning days are considered (Figure 4D): indeed, compared to adult mice, middle-aged mice used less spatial strategies to complete the task (age effect [*F*_(1,32)_ = 23.116, *p* < 0.0001]). Besides, when mice are under a PEGB-enriched diet, they used more spatial strategies than mice under a control diet (diet effect [*F*_(1,32)_ = 10.345, *p* < 0.01]).

### Effects of Age and PEGB-Enriched Diet on Hippocampal and Striatal Gene Expression

**Figure 5:**
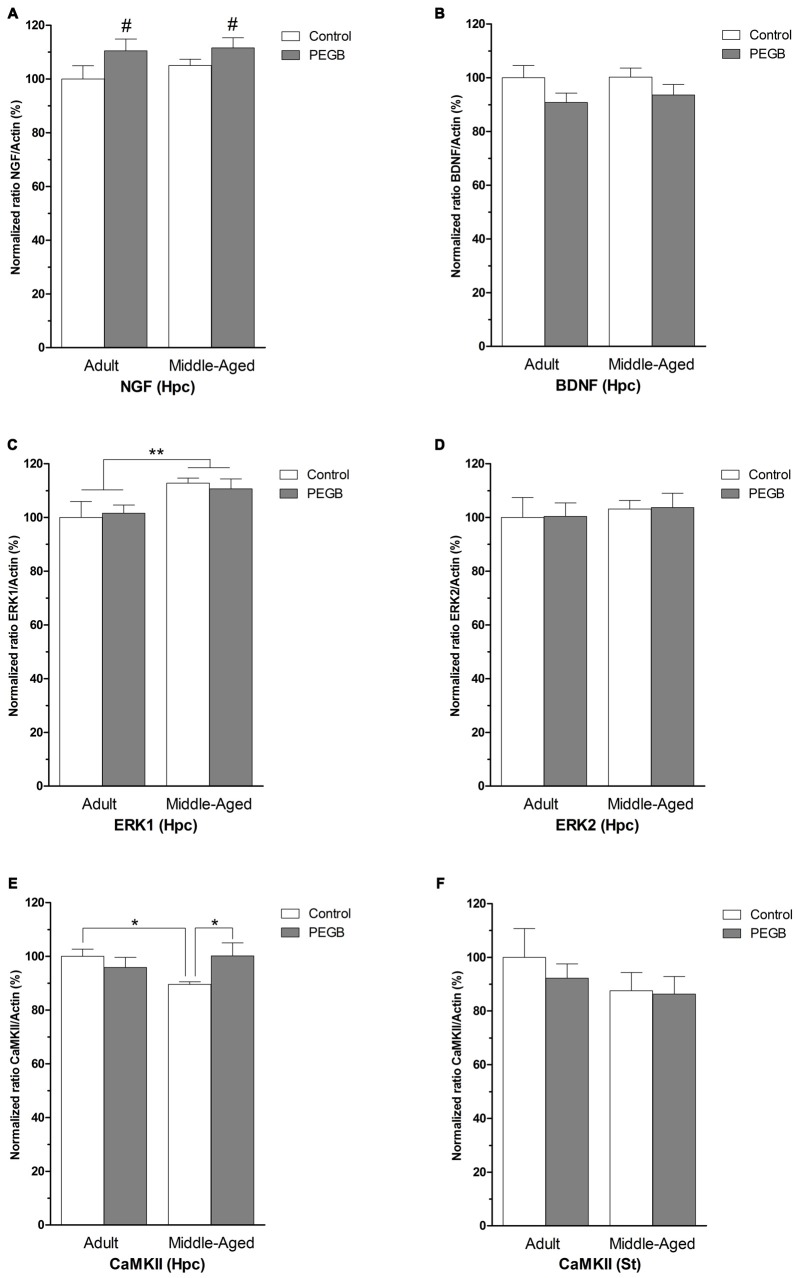
**Effects of PEGB supplementation on hippocampal (Hpc) and striatal (St) plasticity-related gene expression. (A,B)** *NGF* mRNA expression increases significantly in the hippocampus of supplemented animals (diet effect ^#^*p* < 0.05) but no differences induced by age or diet are found in hippocampal brain derived neurotrophic factor *(BDNF)* mRNA levels. **(C,D)** Hippocampal *ERK1* mRNA levels are increased with age but not with polyphenols (age effect **p < 0.01). No changes in hippocampal *ERK2* levels are induced by age nor diet. **(E,F)** Age decreases *CaMKII* mRNA levels in the hippocampus which are restored by the PEGB-enriched diet (*p < 0.05). However, no differences are observed in the striatum (*n* = 6–10 per group).

To study the neurobiological correlates associated with the age-induced spatial deficits and the polyphenol-induced behavioral improvements, the mRNA levels of proteins involved in synaptic plasticity were measured (Figure 5). In the hippocampus a significant increase in *NGF* mRNA levels was observed both in adult and middle-aged mice fed with PEGB-enriched diet (*F*_(1,24)_ = 4.645, *p* < 0.05) without being affected by aging (age effect [*F*_(1,24)_ = 0.604, n.s.]) (age × diet effect [*F*_(1,24)_ = 0.239, n.s.]) (Figure 5A). However, no effect of age or diet was found on the hippocampal *BDNF* mRNA levels (Figure 5B). The ANOVA on *ERK1* mRNA levels revealed an age effect (*F*_(1,25)_ = 7.806, *p* < 0.01) with an increased expression in middle-aged animals (Figure 5C) whereas no effect of age or diet was observed on the *ERK2* mRNA expression (Figure 5D). Moreover, the hippocampal *CaMKII* mRNA expression is decreased in middle-aged mice and could be offset by a supplementation with polyphenols. The ANOVA on hippocampal *CaMKII* expression revealed an age × diet interaction (*F*_(1,28)_ = 4.877, *p* < 0.05) and the *post hoc* Fisher PLSD test showed a significant difference between the mRNA levels observed in middle-aged control mice and those of the adult control mice (*p* < 0.05), and those of the middle-aged supplemented mice (*p* < 0.05) (Figure 5E).

In order to verify the non-involvement of the striatum in the use of spatial strategies, the expression profile of the same mRNAs was assessed. However, in our conditions, striatal *NGF* and *BDNF* mRNA levels were too low to be quantified (data not shown) and no difference in the levels of striatal *CaMKII* mRNA expression was observed showing that the changes observed in the hippocampus were specific to this structure (Figure 5F).

## Discussion

In the present study, we have shown for the first time that a supplementation with a PEGB ameliorates the profile of spatial navigation learning in the Morris Water maze and by this way slightly improves memory of middle-aged mice. Indeed, we evidenced with the categorization of navigation strategies that middle-aged mice take more time to adopt spatial strategies to solve the Morris water maze task. Besides, a supplementation in polyphenols can facilitate the use of spatial strategies by adult and middle-aged mice. Moreover, these learning impairments are associated with decreased hippocampal *CaMKII* mRNA levels whereas performance improvements can be linked to the restoration of hippocampal *CaMKII* mRNA levels and to an increased hippocampal *NGF* expression with the PEGB supplementation.

The Morris water maze is considered as a test of spatial learning and memory (Morris et al., 1982; Morris, 1984) but this task can also be solved by alternative non-spatial strategies. Consistent with previous reports (Morris, 1984; Wolfer and Lipp, 2000; Janus, 2004; Ruediger et al., 2012) adult control mice completed the task using different search strategies along the learning phase, starting from non-spatial strategies, then intermediate strategies like “focal incorrect” and “repeated incorrect” to switch progressively to spatial strategies (notably focal search around the platform and direct swim to the platform). The increase of the use of spatial strategies evolves continuously but not linearly over the trials as mice may alternate non-spatial and spatial strategies over consecutive trials before using almost exclusively spatial strategies. Moreover, we found that this navigation pattern evolution is influenced by age and diet. It is known that the Morris water maze task is particularly sensitive to cognitive deficits related to aging (Frick et al., 1995; Lindner, 1997; Wolff et al., 2002; de Fiebre et al., 2006). Indeed, age would specifically affect spatial allocentric and sequential egocentric strategies in mice (Fouquet et al., 2011). In this study, we have demonstrated for the first time that the evolution from non-spatial to spatial strategies during learning that is particularly delayed with age is promoted with a supplementation in polyphenols.

The analysis of the navigation strategies allows a more thorough knowledge on the acquisition of spatial memory and permits to put on the fore subtle learning differences. Here, longer escape distance to reach the platform traveled by middle-aged mice does not reflect a constant random search of the entire surface area of the pool, which would have indicated a complete lack of spatial learning abilities. It rather shows that some middle-aged mice rely on persistent performance of an alternative strategy that is successful to reach the escape platform but that could appear less efficient if the conditions change. Thus middle-aged mice took more trials to switch to spatial strategies; indeed, their first use of spatial strategies would appear later and most of them would not exclusively use spatial strategies even on the last training day. Indeed, the adoption of spatial strategies is already compromised in 12-month old mice (Gil-Mohapel et al., 2013). In the current study, we used 16–18 month old mice as middle-aged mice as they represent a suitable model with mild cognitive deficits for a 2-month nutritional approach (Pepeu, 2004; Bonhomme et al., 2014).

Here, we show that the PEGB can improve spatial learning in both adult and middle-aged mice. Unlike the middle-aged control mice, middle-aged mice that were fed the PEGB-enriched diet learned as quickly as adult control mice. The extract supplementation could thus prevent from the occurrence of age-related learning deficits. The different families of polyphenols present in this extract are known to be effective on cognitive functions in particular flavanols (van Praag et al., 2007; Rendeiro et al., 2013a), anthocyanins (Rendeiro et al., 2009, 2013a; Shan et al., 2009; Gazova et al., 2013) and resveratrol (Abraham and Johnson, 2009; Dal-Pan et al., 2011; Harada et al., 2011; Kodali et al., 2015). However, this is the first nutritional intervention with a mix of different polyphenols at low doses that shows a rescue effect on those specific memory deficits.

In this study, it could be hypothesized that hippocampal impairments in middle-aged mice impede the use of spatial strategies: middle-aged mice would thus solve the task using preferentially striatum-dependent strategies (Packard and McGaugh, 1996; Devan et al., 1999; Colombo et al., 2003; Kelleher et al., 2004; Teather et al., 2005). Indeed, when the hippocampus is altered, the spatial navigational strategies may not be possible or too costly. On the contrary, polyphenols could preserve the involvement of the hippocampus so that hippocampus-dependent strategies are promoted and age-related deficits reduced.

The neurobiological basis for differences in navigation patterns is not fully understood but it could be hypothesized that differential activations of the hippocampus and the striatum during the learning phase could explain this evolution. Indeed, evidence supports the view that the hippocampus and the striatum act in parallel during the acquisition of various tasks (Colombo et al., 2003; Gazova et al., 2013). Indeed, sustained hippocampal expression of Fos is found in mice and rats that predominantly use allocentric strategies (Passino et al., 2002; Colombo et al., 2003) and c-Jun-immunoreactive cells were observed in the striatum of rats that acquired a cued task (Teather et al., 2005). Phosphorylated CREB immunoreactivity (pCREB-IR) is also increased in the hippocampus of rats that use an allocentric spatial strategy to solve a radial arm maze task, whereas pCREB-IR is increased in the striatum of rats that use an egocentric response strategy (Colombo et al., 2003).

The hippocampus dysfunction during aging could particularly be due to reduced persistence and magnitude of long-term potentiation in hippocampal neurons (Barnes, 1979; Barnes and McNaughton, 1985; Deupree et al., 1991; Barnes et al., 1992; Moore et al., 1993; Geinisman et al., 1995; Rosenzweig et al., 1997; Calhoun et al., 1998; Smith et al., 2000). Polyphenols have been shown to impact on different neuronal signaling pathways involved in synaptic plasticity and long term potentiation (Williams and Grayer, 2004; Schroeter et al., 2007; Vauzour et al., 2007; Rendeiro et al., 2009) and particularly on the modulation of specific signaling pathways involved in learning and memory: our results are partially consistent with these studies. Indeed, *CaMKII* expression that decreases during aging can be recovered by an 8-week polyphenol supplementation. This effect could be attributed to the flavanols, provided mainly from grapes, because a catechin supplementation in aged mice has been shown to restore hippocampal expression level of CaMKII (Abraham and Johnson, 2009) whereas a blueberry supplementation, richer in anthocyanins, failed to restore CaMKII activation in aged rats (Rendeiro et al., 2009). Contrary to what might have been expected, we did not observe modifications in *ERK2* mRNA expression, whereas there was an increase in *ERK1* mRNA expression in middle-aged mice whatever the diet. No modification in mRNA or total protein levels have been described up to now. However, the activation of ERK1/2 has been shown to decrease with aging and to be normalized with polyphenols from blueberry (Rendeiro et al., 2009, 2013a).

Moreover, despite a nutritional approach dealing with low doses of polyphenols, the PEGB used in the present study also permitted to slightly increase hippocampal *NGF* mRNA expression in both adult and middle-aged mice. Such an increase in NGF expression has previously been associated with an improvement of memory performance particularly in aged rodents (Deupree et al., 1991; Woolf et al., 2001). It could thus be hypothesized that the effects of the polyphenols are supported by this *NGF* mRNA level increase (De Nicoló et al., 2013) but it may not be the unique factor underlying these effects. However, no modification in *BDNF* mRNA expression has been observed after an 8-week supplementation in polyphenols although a decrease in BDNF and pro-BDNF mRNA and proteins levels have been reported during normal aging (Phillips et al., 1991; Michalski and Fahnestock, 2003; Peng et al., 2005; Calabrese et al., 2013) and an increase in BDNF levels could have been observed as previously (Rendeiro et al., 2013a).

In order to evidence the differential activation of the hippocampus and the striatum during learning and to determine how polyphenols could impact on the choice of the used strategy, we have also evaluated gene expression in the striatum, focusing on *BDNF, NGF* and *CaMKII* expressions. As *NGF* and *BDNF* mRNA expressions were too low to be quantifiable in the striatum, their modulation may not be responsible for the observed behavioral effects. However, unlike in the hippocampus, *CaMKII* expression in the striatum was not modulated by age nor by polyphenols. These results suggest that the modulation of *CaMKII* expression is specific to the hippocampus and could be linked to the effect of age and polyphenols on the use of “spatial” strategies.

It would be interesting to know how polyphenols precisely act to modify gene expression. It has been established through the use of autoradiography, that specific polyphenol binding sites for resveratrol exist throughout the rat brain (Han et al., 2006). The action of various polyphenols and resveratrol analogs could be mediated by the activation of common “receptor” binding sites particularly enriched at the level of the cellular plasma membrane in the rat brain. It is though unclear what the molecular nature of those receptors might be (Barco et al., 2006). It has also been reported that the effects of resveratrol could also be linked to its interaction with sirtuins (Borra et al., 2005; Donmez et al., 2010; Sadowska-Bartosz and Bartosz, 2014). Besides, van Praag (2009) has proposed the existence of specific membrane receptors for flavonoids.

The present results show that learning alterations in the early stage of aging can be overcome with a nutritional intervention with polyphenols. Our study focused on spatial learning in mice but other potential effects of dietary polyphenols on other kinds of memory must be studied more thoroughly. Thus, polyphenols are potential food nutrients that can help in the prevention of the age-related cognitive decline (Impey et al., 2004; Andres-Lacueva et al., 2005; Schroeter et al., 2007; Shukitt-Hale et al., 2008; Abraham and Johnson, 2009; Rendeiro et al., 2009; Dal-Pan et al., 2011; Harada et al., 2011; Kean et al., 2015) and our results suggest that they can play an important role for memory early in life. Furthermore, future investigations are still needed to better determine how polyphenols act at the molecular level to modulate gene expression, which potentially leads to improved cognitive performance. As preventive strategies for healthy aging are being developed, their definition must take into account environmental factors such as nutrition to promote the maintenance of a satisfactory cognitive state in elderly subjects and to avoid -or at least delay—any evolution towards memory loss and dementia (Gómez-Pinilla, 2008; Joseph et al., 2009; van Praag, 2009; Murphy et al., 2014).

## Author Contributions

JB, DG, SL, VP and PL conceived and designed the experiments; JB, LS, SA and PL performed the experiments; JB, PL and SA analyzed the data; JB and PL wrote the manuscript.

## Conflict of Interest Statement

The authors declare that the research was conducted in the absence of any commercial or financial relationships that could be construed as a potential conflict of interest.

## Supplementary Material

Supplementary Figure 1 **Swim path efficiency during spatial learning.** Swim path efficiency is only affected by age but PEGB-enriched diet tends to improve learning (age effect: ***p* < 0.01; diet effect: *p* = 0.0828; age × diet effect: *p* = 0.0547 by 3-way ANOVA. *n* = 9–11 per group).
