# Supplementary figures and images for: Dietary Polyphenol Supplementation Prevents Alterations of Spatial Navigation in Middle-Aged Mice
Source: Front Behav Neurosci. 2016 Feb 9;10:9. doi: 10.3389/fnbeh.2016.00009 (PMC4746350; doi:10.3389/fnbeh.2016.00009)

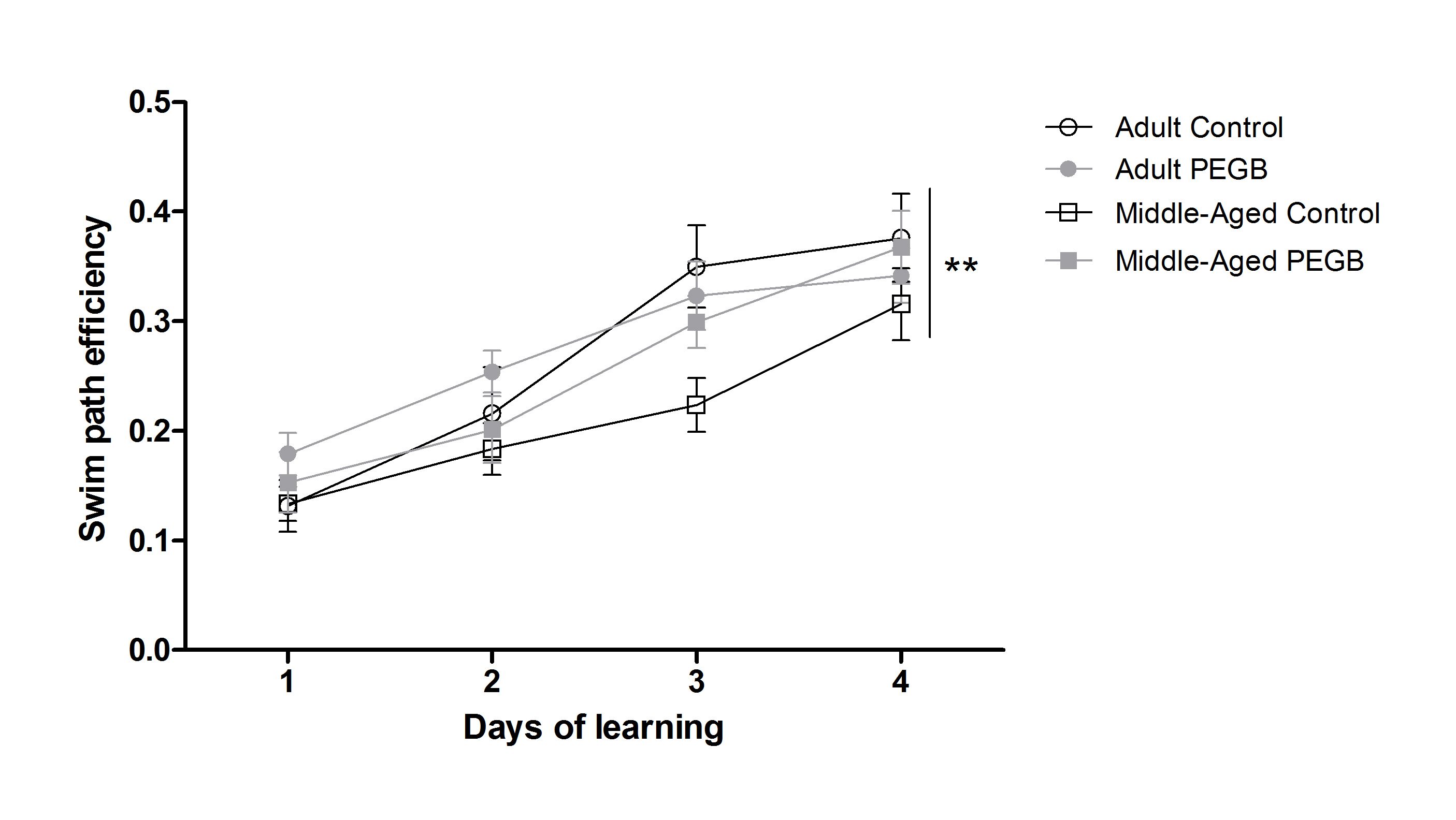

Supplement: Supplementary Figure 1 — Swim path efficiency during spatial learning. Swim path efficiency is only affected by age but PEGB-enriched diet tends to improve learning (age effect: **p < 0.01; diet effect: p = 0.0828; age × diet effect: p = 0.0547 by 3-way ANOVA. n = 9–11 per group). [file Image_1.jpeg]
